# The relationship between gender role and attitude toward menstruation with female sexual function: A cross-sectional study

**DOI:** 10.18502/ijrm.v13i10.7769

**Published:** 2020-10-13

**Authors:** Najme Mokhtari, Azadeh Salavati, Elham Azmoude

**Affiliations:** ^1^Student Research Committee, Torbat Heydariyeh University of Medical Sciences, Torbat Heydariyeh, Iran.; ^2^Department of Midwifery, School of Nursing and Midwifery, Neyshabur University of Medical Sciences, Neyshabur, Iran.

**Keywords:** Menstruation, Gender roles, Sexual behavior.

## Abstract

**Background:**

Although female sexual function can be affected by many psychological, interpersonal, and sociocultural factors, limited studies have focused on the relationships between this construct with some concepts such as women's attitudes toward menstruation and perception from their gender role.

**Objective:**

To investigate the association between gender roles and attitude toward menstruation with sexual function among Iranian reproductive women.

**Materials and Methods:**

An observational cross-sectional study was carried out on a group of 164 Iranian women referred to the health centers of Torbat Heydariyeh, between August and December 2018. All eligible women filled the female sexual function index (FSFI), Bem Sex Role Inventory, and Menstrual Attitude Questionnaire.

**Results:**

The result showed that the total FSFI score was significantly higher in masculine and androgynous gender role groups than women with undifferentiated gender role (p = 0.014, and p = 0.012, respectively). Nevertheless, androgynous women had better sexual arousal than undifferentiated women (p = 0.013). Significant reverse correlations were observed between the perception of menstruation as a debilitating, bothersome, and predictable event with a total score of FSFI and all subscales except lubrication (p = 0.001). In contrast, there was a positive relationship between denial of any effects of menstruation with total FSFI and all its sub-scores except lubrication (p = 0.001).

**Conclusion:**

This study highlighted the role of masculinity and androgyny gender role stereotypes and menstrual attitude in the sexual function of heterosexual women. Future studies are needed to explain how these psychological contexts contribute to different aspects of women's sexuality.

## 1. Introduction

Sexual function is an important dimension of life and has significant effects on the emotional and physical health of an individual (1, 2). Female sexual dysfunction can be affected by a number of biological, psychological, social, and medical factors, the identification of which helps in understanding the multidimensional nature of human sexuality (3). For instance, menstruation as an important symbol of sexual maturity and fertility potential of women had important effects on the different aspects of women's functioning (4, 5). In this regard, although almost all women in the world experience menstruation, their attitudes toward and perceptions of this global phenomenon are different (5, 6). For instance, in a survey of high school students in Iran, only 12.3% of participants believed that menstruation is a natural event (7). In contrast, in the study by Anjum in Pakistan, 87.6% of the females regarded it as a natural process of cleaning (8).

Although numerous scientific studies have focused on studying the sexual behavior throughout the menstrual cycle, there is a paucity of studies on the relationship between the attitudes toward menstruation and sexual behaviors (9, 10). Indeed, because menstruation and sexuality are related to the same part of the body; women with a negative attitude to menstruation may have a negative light to sexuality as well (4). In this regard, some researchers have found that women's attitudes toward menstruation play a role in the perception of their body, gender identity, and sexuality (4, 6).

Another variable that may influence human sexual behavior is self-perceptions of gender role (11). The gender role is defined as the experience or the degree to which one considers themself as masculine and/ or feminine (12). The relationship of this concept with some aspects of human sexuality has been documented. For instance, based on some researches, androgynous subjects report higher levels of sexual self-esteem and greater sexual satisfaction than sex-typed or undifferentiated individuals (11). Conversely, a recent study has shown no significant relationship between gender role and sexual assertiveness in Iranian women (13).

Despite the importance of identifying the effects of women's menstrual attitude and gender role self-perceptions on various aspects of sexuality, most studies in this area have been conducted in Western countries. Therefore, in order to address this gap in the knowledge, the present study was undertaken to find the relationships between attitude toward menstruation and attitude toward gender role with female sexual function.

## 2. Materials and Methods

This cross-sectional study was conducted in all urban healthcare centers in Torbat Heydariyeh between August and December 2018. Torbat Heydariyeh is located in eastern Iran, in The Razavi Khorasan Province. Based on a pilot study, using the correlation coefficient formula, considering the power of the study of 80% and an error of < 5%, a final sample size of 160 subjects qualified for this study.

The inclusion criteria of the study included women who were currently married, sexually active, had no chronic illness (e.g., mental illness, cardiac disease, etc.), and absence of identified sexual dysfunction in the male partner. Women who were divorced, widowed, pregnant or lactating, and those with stressful events during three months before the start of the study were excluded.

Data were collected after a visit at the health centers on the course of study. Those who agreed to take part in the study filled out the questionnaire after they were informed about the purpose and procedure of the study. The survey included demographics, menstrual and reproductive characteristics form, female sexual function index (FSFI), Bem Sex Role Inventory, and Menstrual Attitude Questionnaire.

The demographic information included participant's age, educational level, and employment status. Menstrual and reproductive variables included menarche age, duration of a menstrual cycle and a menstrual bleeding, painful menstruation, dysmenorrhea severity, as well as parity.

### Female sexual function index (FSFI)

The presence of sexual dysfunction over the course of four weeks was evaluated using the FSFI. This scale included 19 items covering six aspects of female sexuality: orgasm, desire, arousal, lubrication, pain and satisfaction. The scale score varied between 2 to 36, with a higher score signify a more better sexual function in past weeks. The total score was calculated by summing the scores of the six subscale (14). The Persian version of this scale has an acceptable range of internal consistency (with a Cronbach's alpha value between 0.72 and 0.90), as reported in a study by Fakhri *et al*. (15). The study results indicated a good internal consistency with coefficients ranging from 0.75 to 0.92 for all subscales and overall.

### Bem sex role inventory

The bem sex role inventory was used to determine a gender role identity. This instrument is a 60-item self-report measure (20 masculine, 20 feminine, and 20 neutral items). It is a 7-point Likert instrument, that ranges from 1 “never/almost never true" to 7 “always/almost always true". Based on the scale, the subjects divide into four different groups: masculine, feminine, androgynous (high degree of both masculine and feminine attributes), and undifferentiated (a decrement in both masculine and feminine traits) (16). The validity and reliability of the Persian version of the questionnaire was confirmed in a study conducted by Alavi and colleagues (Cronbach's alpha coefficients ≥ 0.7 in both masculinity and femininity subscales) (17). The reliability coefficients for masculinity and femininity subscales in the present study were 0.77 and 0.84, respectively.

### Menstrual attitude questionnaire 

This scale was utilized to measure women's attitudes toward menstruation. The questionnaire consisted of 33 items to which each items was responded on a 7-point Likert scale (strongly disagree to strongly agree). It contains five subscales: Menstruation as a Debilitating Event (the most negative item), a Bothersome Event (less negative), a Natural Event (the most positive item), an Event whose can be Anticipated and Predicted and Denial of any Effects of Menstruation (18). The questionnaire has shown to have a good validity among Iranian population in a previous study (7). The present results showed that the Cronbach's alpha coefficients for five subscales of this scale were between 0.75 and 0.94.

### Ethical consideration

The study was approved and authorized by the Ethical Committee of Torbat Heydariyeh University of Medical Science (code: IR.THUMS.REC.1396.16). The purpose of the study was explained to all women. A written consent form was obtained from the women who were willing to participate. They were confident that participation was completely voluntary and had no effect on their routine care.

### Statistical analysis

Data were analyzed using the Statistical Package for the Social Sciences (SPSS) version 16. The normality of continuous variables was assessed using the one-sample Kolmogorov-Smirnoff test. To study the relationship among variables, Spearman's correlation coefficients were applied. The differences were tested using the Kruskal-Wallis and the post-hoc tests using the Mann-Whitney U-test. A p-value < 0.05 was considered as statistically significant.

## 3. Results

The final study population comprised of 164 women. The mean age of the participants was 27.78 ± 6.59 (range 18.0-45.0) yr and mean parity was 1.13 ± 1.16 (range 0.0-5.0). The mean menarche age was also 12.71 ± 1.63 (range 10.0-17.0) and the mean length of menstrual cycle was 28.10 ± 4.05 (range 20.0-40.0) days. The majority had higher education (51.2%) and were housewife (75.3%). Also, primary dysmenorrheawas experienced by 28.3% of the participants and secondary dysmenorrhea by 7.8%. In total, 62.8% of the women stated that they had a regular menstrual cycle. The mean ± SD total score of FSFI was 27.34 ± 4.50.

**Figure 1:**
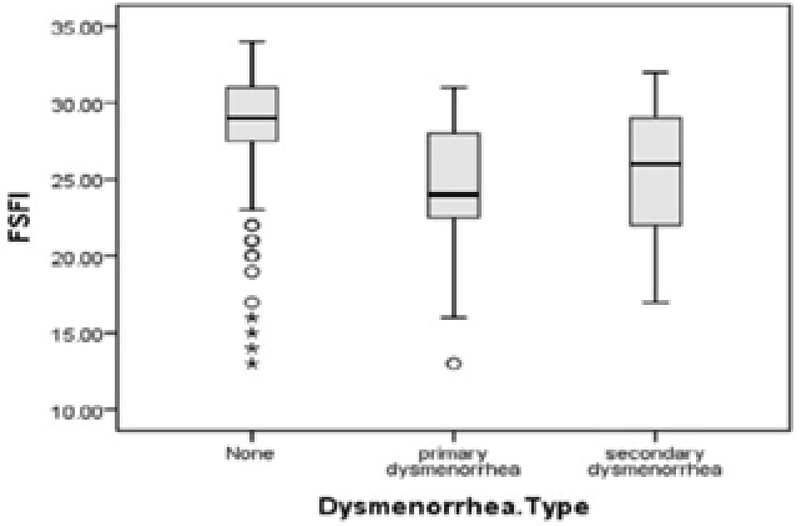
Boxplot of the FSFI score distribution by dysmenorrhea type.

**Figure 2:**
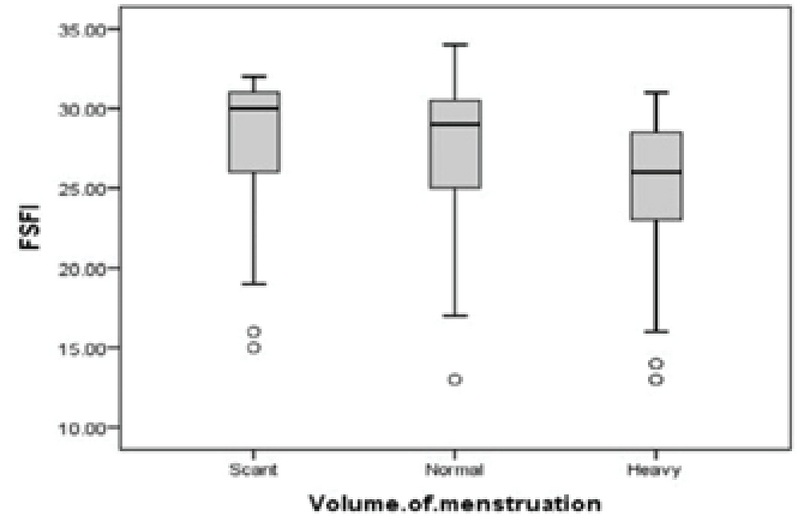
Boxplot of the FSFI score distribution by volume of menstruation.

The analysis of the data revealed no significant relationships between the mean age and body mass index with sexual function score (p = 0.055 and p = 503, respectively). The women with primary and secondary dysmenorrhea had lower levels of FSFI score than women without dysmenorrhea (p = 0.001) (Figure 1). Additionally, the mean score of FSFI was higher in women who perceived volume of menstruation as scant than in those who considered it as normal or severe (p = 0.003) (Figure 2). In this study, 9.8% (n = 16) of the participants were in the feminine gender role group, 10.4% (n = 17) in the masculine, 44.5% (n = 73) in the undifferentiated, and 35.4% (n = 58) in the androgynous group.

The results of the Kruskal-Wallis test showed significant differences in the mean score of the total FSFI and the arousal subscale across the gender role types (p = 0.013, p = 0.048, respectively). To compare the differences between groups, the Mann-Whitney U-test was used. The results revealed that individuals with masculine and androgynous gender roles had more total FSFI score than the undifferentiated women (p = 0.014 and p = 0.012, respectively). Nevertheless, androgynous women had more arousal score than the undifferentiated women (p = 0.013). However, there were no significant differences in other subscales of FSFI in terms of gender role types (Table I).

The mean scores on the subscales of menstrual attitude questionnaire ranged from 3.68 ± 1.42 to 5.24 ± 1.01, indicating that the highest and the lowest mean scores belonged to menstruation as a natural event and menstruation as a bothersome event, respectively. There were significant reverse relationships between the perception of menstruation as a debilitating, bothersome, and predictable event with a total score of FSFI and all subscales except lubrication. In contrast, there was a positive relationship between denial of any effects of menstruation with total FSFI and all its sub-scores except lubrication. However, the results showed no significant relationships between the perception of menstruation as a natural event with a total score of FSFI and all subscales (Table II).

**Table 1:** Female sexual function and its domain scores in four gender role types


**Variables**	**Desire**	**Arousal**	**Lubrication**	**Orgasm**	**Pain**	**Satisfaction**	**Total FSFI**
**Feminine**	4.08 ± 1.26	4.74 ± 1.39	4.70 ± 1.08	4.77 ± 1.24	4.55 ± 1.37	4.60 ± 1.19	27.46 ± 5.88
**Masculine**	4.51 ± 1.30	4.81 ± 0.93	4.71 ± 0.47	4.87 ± 0.88	5.12 ± 0.68	4.91 ± 0.92	28.96 ± 4.38
**Androgynous**	4.34 ± 0.85	4.83 ± 0.92	4.63 ± 0.54	4.85 ± 0.82	4.97 ± 0.97	4.40 ± 0.92	28.05 ± 3.92
**Undifferentiated**	3.95 ± 0.95	4.40 ± 1.05	4.43 ± 0.78	4.52 ± 0.97	4.75 ± 0.79	4.31 ± 1.12	26.37 ± 4.51
**P-value**	0.066	0.048	0.248	0.126	0.051	0.115	0.013
Data presented as Mean ± SD. Kruskal-Wallis test, p-value < 0.05 was significant FSFI: Female sexual function index							

**Table 2:** Bivariate correlations between attitudes toward menstruation with female sexual function and domain scores


**Variables**	**Desire**	**Arousal**	**Lubrication**	**Orgasm**	**Pain**	**Satisfaction**	**Total FSFI**
**As a debilitating event**	-0.267${}^{**}$	-0.388${}^{**}$	-0.138	-0.393${}^{**}$	-0.235${}^{*}$	-0.341${}^{**}$	-0.380${}^{**}$
**As a bothersome event**	-0.297${}^{**}$	-0.281${}^{**}$	-0.112	-0.326${}^{**}$	-0.250${}^{**}$	-0.341${}^{**}$	-0.370${}^{**}$
**As a natural event**	0.016	r = 0.058	0.138	r = 0.148	0.056	0.031	0.051
**As an event that can be anticipated**	-0.407${}^{**}$	-0.461${}^{**}$	-0.121	-0.348${}^{**}$	-0.409${}^{**}$	-0.304${}^{**}$	r = -0.452${}^{**}$
**Denial of the effects of menstruation**	0.302${}^{**}$	0.403${}^{**}$	0.000	r = 0.373${}^{**}$	0.441${}^{**}$	0.342${}^{**}$	r = 0.430${}^{**}$
Spearman result, *Correlation is significant at the 0.05 level (2-tailed). *p < 0.01, **p < 0.001 FSFI: Female sexual function index							

## 4. Discussion

The present study was intended to determine the relationship between the attitude toward menstruation and the attitude toward gender role with female sexual function. The results of the study showed the women who were classified as masculine and androgynous reported significantly higher levels of sexual function than those with undifferentiated gender roles. In the between-groups comparison, we did not notice any significant differences in desire, lubrication, orgasm, satisfaction, and pain expect arousal. Based on the literature, it has been stated that the gender roles, as a culturally constructed phenomenon, affect all aspects of life, especially how one behaves sexually (19, 20).

In line with these findings, Fischer and colleagues in their surveys carried out among undergraduates student in USA reported that androgynous and sex-typed individuals experienced more intimacy than undifferentiated individuals (21). In another survey in clinic and non-clinic couples, Safir *et al* also reported that if one or both partners were androgynous, the couples' sexual function was better than when both of them were sex-typed (22). Additionally, in another study, women with masculine and androgynous gender roles reported a greater number of sexual activities than feminine and undifferentiated individuals (23).

Indeed, some of the attributes associated with positive sexual experiences such as self-reliance, activeness, assertiveness, open-mindedness, independence, and leadership, in the subjects with masculine and androgynous characteristics may have significant effects on the promotion of sexual function (24). In addition, according to the pieces of evidence, androgynous subjects have a more comfortable attitude toward sexual activity, which may lead to better sexual function (25). Androgynous individuals are also capable to express a masculine or feminine response depending on the situation, which may moderate sexual function (26). Nevertheless, most women have been taught that women are expected to be submissive in sexual interactions (27). Therefore, based on this study, it is possible that women with a masculinity gender role are less affected by negative sexual script.

The study of Arbanas and coworker suggest that masculinity is an important factor for sexual functioning in heterosexual women (28). Generally, it is well established that the masculinity, rather than femininity, is related to a wide range of positive aspects of sexuality (28, 29). In contrast to this study, Spencer reported no differences between androgynous and non-androgynous subjects with respect to their levels of sexual dysfunction. One of the major possibilities for explaining this discrepancy was the usage of different instruments for measuring sexual function (30).

Another notable finding was that perceptions of menstruation as being bothersome and debilitating, and predictable events were associated with decreasing sexual desire, arousal, orgasm, satisfaction, and increasing pain during intercourse. On the other hand, the belief that menstruation has no effect on the individual was associated with more sexual function and all dimensions score. In contrast, the perception of menstruation as a natural event was not associated with sexual function. Since, both sexuality and menstruation are related to the same area of the body, women who have believed that menstruation is bothersome and debilitating may have similar reactions toward sexuality. In addition, these results show that negative attitude toward menstruation were more associated with women's sexuality than positive attitude. Similarly, Schooler and colleagues, in their study on undergraduate women, reported that menstrual shame was associated with less sexual experiences and more sexual risk-taking behavior (31).

Rempel and co-workers, also suggested the women who were more comfortable and had accepting attitude toward menstruation were more inclined to feel comfortable with their own sexuality (4). Thus, as menstruation plays an important role in different aspects of a woman's life, it is crucial that society and women's attitudes toward this cultural phenomenon as a natural part of life be corrected, which further suggests providing the appropriate background and prerequisites.

## 5. Conclusion

In summary, this study revealed that gender role as well as menstrual attitude was related to female sexual functioning. However, further studies are needed to focus on how these psychological contexts contribute to the different aspects of sexuality with a more comprehensive profile. Moreover, there are several limitations in this study. The cross-sectional design of the study can significantly limit its capability in explicating the causal relationships between variables. A qualitative design research study may help describe the complex relationship between the studied contexts. Despite these limitations, this study clarifies some psychological factors that may affect female sexual function. This study may help design effective interventions to develop a healthy attitude toward menstruation and thus improve the female sexual function.

##  Conflict of Interest 

The authors report no conflicts of interest.
